# Adherence to the Mediterranean Diet in Pregnancy and Its Benefits on Maternal-Fetal Health: A Systematic Review of the Literature

**DOI:** 10.3389/fnut.2022.813942

**Published:** 2022-03-31

**Authors:** Ana Zaragoza-Martí, Nuria Ruiz-Ródenas, Irene Herranz-Chofre, Miriam Sánchez-SanSegundo, Verónica de la Cruz Serrano Delgado, Jose Antonio Hurtado-Sánchez

**Affiliations:** ^1^Department of Nursing, University of Alicante, Alicante, Spain; ^2^Alicante Institute for Health and Biomedical Research (ISABIAL-FISABIO Foundation), Alicante, Spain; ^3^Servicio de Ginecología y Obstetricia, Hospital General Universitario de Elche, Elche, Spain; ^4^Department of Health Psychology, University of Alicante, Alicante, Spain; ^5^Servicio de Ginecología y Obstetricia, Hospital Marina Salud, Denia, Spain

**Keywords:** Mediterranean diet, pregnancy, adherence, benefits, offspring

## Abstract

**Introduction:**

Pregnancy is a transcendent period for the mother and the fetus, characterized by an increase on energy requirements. Mediterranean diet (MD) is considered a healthy eating pattern that can provide the nutritional requirements of pregnancy and protect from the development of obstetric pathologies.

**Objective:**

To know the relationship between adherence to the MD and its maternal-fetal benefits.

**Methodology:**

A systematic review was conducted by identifying articles in the *PubMed* and *Cochrane* databases. The publication date of the studies was between 2010 and 2020, and the inclusion criteria established were that the articles were written in English and Spanish and were accessible in full text. Studies concerning assisted reproduction, gene modulation, conference abstracts, systematic reviews, and pilot studies were excluded.

**Results:**

Finally, a total of 14 studies were included in the review. The association between the MD and the reduction of some pathologies of pregnancy, such as gestational diabetes, overweight or obesity, sleep quality, complications of childbirth, urinary tract infections (UTIs), and alterations in fetal growth was demonstrated, as well as perinatal problems, including birth weight, prematurity, gastroschisis, and other childhood problems.

**Conclusion:**

The MD is an optimal diet to consume during pregnancy.

## Introduction

Women’s nutritional habits during pregnancy are considered one of the most important aspects for maternal-fetal health (1). During pregnancy, energy and nutrient needs increase to enable fetal growth and development, and major physiological changes occur, including maternal tissue formation, increased uterine size, mammary gland hypertrophy, and accumulation of fat reserves (1).

The physiological changes at the gastrointestinal level experienced by women during pregnancy (a decrease in gastric and intestinal peristalsis together with an increase in the intestinal absorption capacity of water and nutrients) (2), together with weight gain (2) and the lack of essential micronutrients that can lead to anemia (1) are essential aspects of intervention. Therefore, maternal nutritional status before pregnancy and adequate eating habits are very relevant requirements during this period of the life cycle (1). Maternal weight has been shown to have a significant relationship with fetal growth and development, so it is important to control weight gain to maintain it within a healthy range. According to the *Centers for Disease Control and Prevention* (CDC), women with a normal body mass index (BMI; between 18.5 and 24.9) are recommended to gain between 11 and 15 kg during pregnancy to reduce the risk of obstetric and perinatal complications (3). An increased weight gain during pregnant has been associated with higher risk of instrumented delivery and Cesarean section, gestational diabetes (GD), fetal macrosomia, neural tube defects, postpartum maternal obesity, and even obesity during childhood and diseases in the adult life of the newborn (1). Maternal insufficient weight gain has also been considered as a risk factor for miscarriage, preterm birth, and low birth weight (1). In this sense, it has been found that newborns with low birth weight may have difficulty establishing adequate breastfeeding as well as an increased risk of contracting diseases and developmental delays (3).

The lack of micronutrients during pregnancy has also been linked to the presence of anemia (1). According to data from the World Health Organization (WHO), it is estimated that, in 2011, around 38.2% of pregnant women suffered from anemia, the main cause being iron deficiency (4). Anemia during pregnancy is directly associated with a high risk of preterm birth, low birth weight, maternal mortality (1), and perinatal (5) and neonatal mortality (1).

Given the available evidence of changes affecting women’s health during pregnancy, it is necessary to follow an adequate diet during gestation. The Mediterranean diet (MD) is considered one of the dietary patterns that guarantees a better supply of nutrients. The MD is defined as the traditional dietary pattern found in the early 1960s in Greece, southern Italy, Spain, and other olive-growing countries of the Mediterranean basin (6). The MD use generou amounts of extra virgin olive oil (EVOO) as the main culinary fat and has a high intake of foods of plant origin: fruits, vegetables, legumes, nuts and seeds, and whole unprocessed grains, a moderate intake of fish, poultry, eggs, and dairy products, especially yogurt and cheese, but not butter or cream, and low intake of sweet desserts, red and processed meats (6).

Several previous studies, particularly those reported within the largest MD research project to date, PREDIMED [PREvención con DIeta MEDiterránea (Prevention with MD)] (7), conclude that following an MD is inversely related to overall mortality (8), the incidence of coronary heart disease (9), cardiovascular events (10), and thrombotic stroke (11). It is also inversely related to the incidence of cancer in general (12, 13) (including breast cancer (14) and colorectal cancer (15), as well as to Type 2 diabetes mellitus (in adulthood) (16) or even to hip fractures (17). All these benefits may be favored by the high content of antioxidants of this plant diet, the high fiber content, and the low glycemic load, in addition to the high content of monounsaturated and polyunsaturated fatty acids in relation to saturated ones, as the former reduce cholesterol levels (6).

MD has also positive effects on pregnancy. Numerous studies show that prenatal diet affects maternal-fetal outcomes. Specifically, the St. Carlos Gestational Diabetes Mellitus Prevention Study (18) establishes a direct relationship between high adherence to MD during pregnancy and decreased risk of GD, urinary tract infections (UTIs), prematurity, and low-weight newborns (18). Other research shows more benefits for the mother, as adherence to MD during pregnancy is also associated with better sleep quality throughout pregnancy (19). In addition, MD prevents overweight and obesity during pregnancy, which means that it helps to maintain adequate weight gain (20). Regarding perinatal outcomes, studies conclude that high adherence to MD during pregnancy reduces some cardiovascular risk factors such as high blood pressure and adiposity (21).

Given the available evidence that MD could play a protective role in maternal and fetal health during pregnancy, the aim of this study was to conduct a systematic review of the literature to study the effects of MD during the gestational period.

## Materials and Methods

A systematic review of the literature was carried out, following the PRISMA methodology (22) as a reference. The quality of each of the studies was assessed with the Cochrane Collaboration Risk of Bias (ROB) tool (23), which includes seven items that analyze six domains of bias. This tool classifies the quality of articles into three levels: high, low, or unclear.

The first author (AZ-M) and the second author (NR-R) of the study rated each included article independently, and the discrepancies were resolved through an agreement with the third author (MS-S). Cohen’s kappa statistic was calculated to assess reliability among evaluators for the ROB without elements that assessed the blinding of participants or evaluators, as all studies were rated as high ROB by the two evaluators when all the elements were analyzed. Inter-evaluator reliability was analyzed using Cohen’s kappa statistic, obtaining an intraclass correlation coefficient (ICC) value of 0.8.

### Data Sources

To obtain the documents, electronic searches were carried out in the international databases *PubMed* and *Cochrane Library*.

### Search Strategy

To obtain the documents, electronic searches were carried out during the months of October, November, and December 2020. We used the following keywords transformed into terms MeSH (Medical Subject Heading): “diet, Mediterranean,” “pregnancy,” “treatment adherence and compliance” combined with the Boolean operators AND and OR. Table 1 shows the search strategy used in the databases.

**TABLE 1:** Database search strategy.

Search strategy
#1 (“Mediterranean diet” [Title/Abstract] OR “Diet, Mediterranean” [MeSH Terms])
#2 (Pregnancy [Title/Abstract] OR Pregnancy [MeSH Terms])
#3 (Adherence [Title/Abstract] OR Treatment Adherence and Compliance [MeSH Terms])
#4 #1 AND #2 AND #3

### Inclusion and Exclusion Criteria

The inclusion criteria were: (1) articles accessible in full text, open access, and available abstract; (2) articles written in English or Spanish; (3) articles published within a range of 10 years: between 2010 and 2020; (4) articles that included at least one sample of pregnant women, and (5) that included MD as an outcome.

We excluded articles that analyzed (1) sterility or assisted reproduction techniques; (2) gene modulation studies; (3) duplicate studies; (4) conference abstracts; (5) systematic reviews or meta-analyses; (6) pilot studies.

### Selection of Articles

The selection of articles was made by reading the title and abstract of all articles resulting from the search in *PubMed*, and *Cochrane*. The quality of each study was independently evaluated by two authors, using the Crombie criteria adapted by Petticrew and Roberts. Disagreements were resolved by a third author.

The AXIS (24) critical evaluation tool was used to assess the quality and ROB in cross-sectional studies (Table 2). Randomized clinical trials were assessed using the PEDro tool (25) (Table 3). Finally, both the quality of the cohort studies (Table 4) and the quality of cases and controls were assessed with the Newcastle-Ottawa scale (26) (Table 4). For all scales, agreements were above kappa values of 0.75. The third and last authors (MS-S and AZ-M) rated each included article independently, and discrepancies were resolved by agreement with the second author (JH-S). We also calculated the Cohen’s Kappa statistic to assess interrater realibility. The Cohen’s Kappa provides a level of agreement between two or more raters than is higher than chance. It is considered as moderate or substantial when values are above 0.6 (moderate) or 0.8–1 as substantial agreement (27).

**TABLE 2:** First ten questions of the AXIS tool.

References	1	2	3	4	5	6	7	8	9	10	11	12	13	14	15	16	17	18	19	20
Flor-Alemany et al. (19)	YES	YES	NO	YES	YES	YES	DK/NR	YES	YES	YES	YES	YES	DK/NR	-	YES	YES	YES	YES	NO	YES
Silva-del Valle et al. (20)	YES	YES	NO	YES	DK/NR	DK/NR	DK/NR	YES	YES	YES	YES	YES	DK/NR	-	YES	YES	YES	YES	DK/NR	YES

*Appraisal of Cross-sectional studies AXIS: 1. aims; 2. study design; 3. sample size justification; 4. target reference population; 5. sampling frame; 6. sample selection; 7. non-responders; 8. measurement validity and reliability; 9. risk factors and outcomes. 10. statistics; 11. overall methods; 12. basic data; 13. non-response bias; 14. non-responders; 15. internal consistency results; 16. comprehensive description results; 17. justified discussions and conclusions; 18. limitations; 19. conflict of interest; 20. ethical approval. DK, Doesn’t know; NR, No reply.*

**TABLE 3:** PEDro tool questions.

References	1	2	3	4	5	6	7	8	9	10	11
Assaf-Balut et al. (18)	YES	YES	NO	YES	NO	NO	NO	YES	NO	YES	YES
Assaf-Balut et al. (29)	YES	YES	YES	YES	NO	NO	YES	YES	YES	YES	YES
Al-Wattar et al. (35)	YES	YES	YES	YES	NO	NO	YES	YES	YES	YES	YES
Melero et al. (31)	YES	YES	YES	YES	NO	NO	NO	NO	NO	YES	YES

*PEDro scale: 1. eligibility criteria; 2. subjects were randomly allocated to groups; 3. allocation; 4. the groups were similar at baseline; 5. there was blinding of all subjects; 6. blinding of all therapists who administered the therapy; 7. there was blinding of all assessors who measured at least one key outcome; 8. measures of at least one key outcome were obtained from more than 85% of the subjects initially allocated to groups; 9. all subjects for whom outcome measures were available received the treatment or control condition as allocated or, where this was not the case, data for at least one key outcome was analyzed by “intention to treat”; 10. results between-group are reported for at least one key outcome; 11. the study provides both point measures and measures of variability for at least one key outcome.*

**TABLE 4:** Tool questions Newcastle-Ottawa.

References	1	2	3	4	5	6	7	8
**Cohort studies**								
Chatzi et al. (21)	*	*	-	*	**	*	*	*
Saunders et al. (36)	*	*	*	*	**	*	*	*
Steenweg de Graaff et al. (33)	*	*	-	*	**	-	*	*
Tobias et al. (37)	*	*	-	*	**	-	*	*
Parisi et al. (34)	*	*	-	-	**	*	*	*
**Case-control studies**								
Olmedo-Requena et al. (28)	-	*	*	*	**	-	*	*
Martínez-Galiano et al. (30)	-	*	*	*	**	-	*	-
Cánovas-Conesa et al. (32)	-	*	*	*	**	-	*	*

*Items of Newcastle-Ottawa Scale for cohort studies: 1. representativeness; 2. non-exposed cohort; 3. ascertainment of exposure; 4. outcome; 5. comparability of cohorts; 6. assessment of outcome; 7. follow-up; 8. adequacy of follow-up. A maximum of one star is allocated for each domain within the “Selection” and “Outcome” categories; and a maximum of two stars is allocated for “Comparability”.*

### Extracted Data

Data extraction was performed by the lead review author, taking into account the year of publication (2010–2020), the design and objective of the study, the year in which the study was conducted, the sample size, the mean age of the participants, the country of origin, the outcomes of the interventions, and the conclusions of the studies.

### Synthesis of Results

The results were grouped into two large blocks: (1) maternal outcomes and (2) perinatal outcomes, which were classified according to the description of the concrete benefits of consuming MD during pregnancy. The maternal outcomes were divided into five sub-blocks: (I) Gestational diabetes (GD), (II) Healthy lifestyle, (III) Complications in childbirth, (IV) UTIs, and (V) Fetal growth; whereas the benefits found in newborns, were grouped into four subgroups: (I) Birth weight, (II) Prematurity, (III) Gastroschisis, and (IV) Problems in childhood.

## Results

**FIGURE 1:**
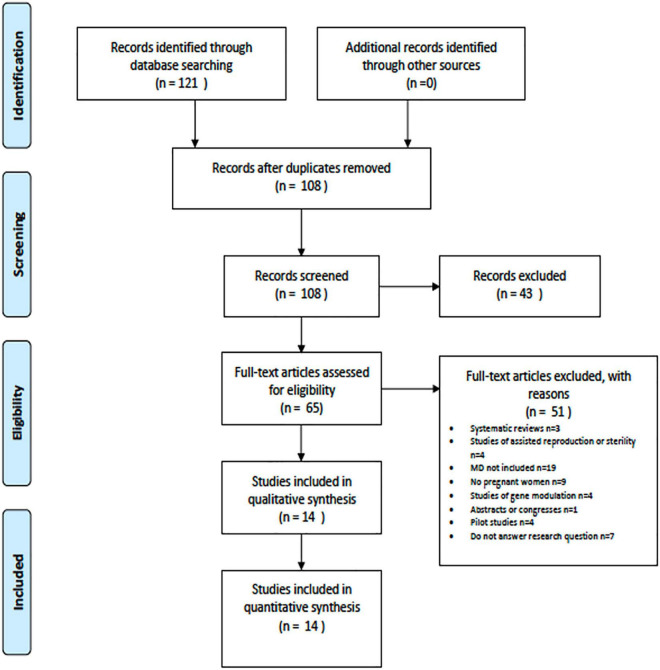
Selection of studies.

A total of 121 articles were identified. After removing the duplicates there were 108 articles. After applying the “2010–2020,” “English” and “Spanish” filters, and all duplicate articles were discarded: 43 articles were deleted. Subsequently, the titles and abstracts were read, and another 51 articles were discarded, according to the inclusion and exclusion criteria set out above. Finally, a total of 14 articles were included in the review (Figure 1).

### Descriptive Data and Types of Studies

Table 5 shows the characteristics of the articles included. Due to the nature of this review, all participants in these studies were pregnant women whose mean age was 31 ± 2.36 years.

**TABLE 5:** Description of the studies.

References	Country	Year	Mean age	Sample n	Objective	Type of study	Dietary Mediterranean data
Assaf-Balut et al. (18)	Spain	2015	32.9	874	To assess the effect of late adherence (>12 week) to an MD pattern based on six dietary objectives (>12 servings/week of vegetables, > 12 servings/week of fruits, < 2 servings/week of juice, 3 servings/week of nuts, > 6 days/week of EVOO intake, and ≥ 40 mL/day) on maternal-fetal complications.	Clinical trial	MEDAS questionnaire
Flor-Alemany et al. (19)	Spain	2015–2017	32.9	159	To explore the association of eating habits and adherence to MD with sleep quality during pregnancy.	Cross-sectional study	FFQ
Silva-del Valle et al. (20)	Spain	2010	30	170	To estimate the degree of adherence to MD in pregnant women in Gran Canaria before pregnancy and in the third trimester, assessing its relationship with weight gain.	Cross-sectional study	FFQ
Chatzi et al. (21)	United States, Crete, and Greece	1999–2012	Mother: 31.5 Son: 6.4	1,566 mother-child dyads	To investigate the associations of maternal adherence to MD early in pregnancy with obesity and cardiometabolic risk in childhood.	Cohorts	Mediterranean diet score (MDS)
Olmedo-Requena et al. (28)	Spain	2010	30.5	1,466	To assess the effect of the level of exposure to MD before pregnancy on the likelihood of developing GD.	Cases and controls	The index developed by Trichopolou et al.
Assaf-Balut et al. (29)	Spain	2015–2016	32.9	874	To assess the effect of an intervention based on MD reinforced with abundant EVOO and nuts (pistachios) on the incidence of GD at 24–28 weeks of gestation.	Clinical trial	MEDAS questionnaire
Al-Wattar et al. (35)	England	2014–2016	31.1	1,138	To assess the effects of a Mediterranean-style diet (supplemented with walnuts and EVOO) and individualized dietary advice on maternal and perinatal outcomes in pregnant women with metabolic risk factors, compared with routine antenatal care.	Clinical trial	FFQ
Martínez-Galiano et al. (30)	Spain	2012–2015	-	533	To quantify the effect of the maternal MD pattern, as well as the intake of EVOO, on the risk of having a low-weight newborn.	Cases and controls	Three index (MEDAS, Trichopoulou, Panagiotakos)
Melero et al. (31)	Spain	2017–2018	Mothes: 33 Childs: 2	703 mother-child dyads	To assess whether MD supplemented with EVOO and pistachios during pregnancy induces child health benefits during the first 2 years of life.	Clinical trial	MEDAS questionnaire
Cánovas-Conesa et al. (32)	Spain	2007–2012	28.1	45	To analyze the association between adherence to MD at the beginning of pregnancy and the risk of gastroschisis.	Cases and controls	FFQ
Saunders et al. (36)	France (Guadeloupe)	2004–2007	31	728	To assess the impact of adherence to MD during pregnancy on fetal growth retardation and preterm delivery.	Cohorts	FFQ
Steenweg-de Graaff et al. (33)	The Netherlands	2006	Mothers: 31.7 Childs: 1.3. 5 y 6	3,062 mothers and 3,104 children	To assess the effects of maternal dietary patterns early in pregnancy on the development of child behavior.	Cohorts	FFQ
Tobias et al. (37)	United States	1991–2001	32	15,245	To determine whether preconception adherence to dietary patterns, including MD, antihypertensive diet, and alternative healthy eating is associated with the risk of GD.	Cohorts	aMED (alternative Mediterranean diet)
Parisi et al. (34)	The Netherlands	2010–2014	32	228	To investigate the association between maternal dietary patterns at the beginning of pregnancy and fetal growth in the first trimester.	Cohorts	FFQ

As for the country of origin, 57.14% (*n* = 8) (18–20, 28–32) of the articles were carried out in Spain, two studies were carried out in the Netherlands (14.29%) (33, 34), one in England (7.14%) (35), one in France (more specifically on the island of Guadeloupe) (7.14%) (36), one in the United States (7.14%) (37), and, finally, one that included participants from the United States, Greece, and Crete (7.14%) (21). In terms of design, of the 14 studies included, five were cohorts (35.71%) (21, 33, 34, 36, 37), four clinical trials (28.57%) (18, 29, 31, 35), three cases and controls (21.43%) (24, 30, 32), and two were cross-sectional studies (14.29%) (19, 20).

Finally, adherence to the Mediterranean diet was measured through the MEDAS (Mediterranean Diet Adherence Screener) in 3 studies (18, 29, 31), through a FFQ (Food frequency questionnarire) in 7 studies (19, 20, 31–35), one study through the Mediterranean diet score (MDS) (21), another study through the index created by Trichopoulou (28) and another study with the aMED (alternative Mediterranean diet) (37). In addition there was a study that evaluated the Mediterranean diet with three indices, the MEDAS, the one created by Trichopoulou and the one created by Panagiotakos (30).

### Effect of Mediterranean Diet in Pregnant Women

Table 6 shows the results of the studies included in the review regarding maternal benefits.

**TABLE 6:** Maternal outcomes.

References	Intervention	Outcomes	Conclusion
Assaf-Balut et al. (18)	The women of the intervention group attended two group sessions where they were instructed to increase their consumption of EVOO and nuts and received 10 l of EVOO and 2 kg of pistachios in each session. The control group received basic dietary guidelines and was told to limit all types of fat consumption (including < 3 servings/week of walnuts and < 40 ml/day of EVOO). To assess lifestyle and diet, the semi-quantitative diabetes nutrition, and complications trial (DNCT) questionnaires and the MEDAS questionnaires were used, which were administered on three different visits. Blood pressure, height, weight, gestational weight gain, and BMI were assessed and recorded at all three visits.	There was a linear association between high, moderate, and low adherence, and a lower risk of GD (OR = 0.35, 95% CI [0.18, 0.67], *p* = 0.002), urinary tract infections (UTIs) (OR = 0.19, 95% CI [0.07, 0.52], *p* = 0.001), prematurity (OR = 0.30, 95% CI [0.13, 0.72], *p* = 0.007) and low-weight newborns. (OR = 0.36, 95% CI [0.17, 0.77], *p* = 0.009).	High adherence at the end of the first trimester to the six predefined dietary targets is associated with a reduction in the risk of GDG, UTIs, prematurity, and low birth-weight infants.
Flor-Alemany et al. (19)	Eating habits were collected using the food frequency questionnaire (FFQ) designed by Mataix, while the Spanish version of the PSQI was used to assess sleep quality at 16 and 34 weeks of gestation.	The group with higher adherence to MD showed better sleep quality than the group with lower adherence at 16 and 34 weeks of gestation (both, *ps* < 0.05).	Increased adherence to MD, higher intake of fruits and EVOO, and lower intake of red meat and by-products were associated with better sleep quality throughout pregnancy, especially among sedentary women
Silva-del Valle et al. (20)	To assess adherence to MD, a validated 14-item self-administered FFQ was administered. It was completed at two different moments: one at the first prenatal visit and one in the early postpartum period. In addition, BMI was determined at the beginning and in the third trimester, based on the weight and height shown in the medical history.	Women with high initial adherence to MD gained less weight during pregnancy (–1.54 kg, 95% CI [–2.53, –0.56]) than women with poor adherence	High adherence to MD before pregnancy can protect against overweight and obesity during pregnancy. A greater increase in adherence to MD during pregnancy may increase the likelihood of adequate weight gain in pregnancy.
Olmedo-Requena et al. (28)	The study was composed of pregnant women with GD (cases) and without GD (controls). To collect information on the dietary pattern of women, an FFQ was used, in which the frequency of consumption and the average amounts for different food groups during the year before pregnancy were collected, and the index developed by Trichopoulou was used to assess adherence to MD.	High adherence to MD was associated with a reduction in GD (OR 0.61, 95% CI [0.39, 0.94], *p* = 0.028), and very high adherence to MD was associated even more strongly (OR 0.33, 95% CI [0.15, 0.72], *p* = 0.005).	The protective effect of adherence to an MD pattern before pregnancy should be considered as a preventive tool against the development of GD.
Assaf-Balut et al. (29)	The women of the intervention group attended two group sessions where they were instructed to increase the consumption of EVOO and nuts and received 10 L of EVOO and 2 kg of pistachios in each session. The control group received basic dietary guidelines and was told to limit all types of fat intake (including < 3 servings/week of walnuts and < 40 ml/day of EVOO). To assess lifestyle and diet, the semi-quantitative diabetes nutrition and complications trial (DNCT) questionnaires and the MEDAS questionnaires were used, which were administered on 3 different visits. Blood pressure, height, weight, gestational weight gain, and BMI were assessed and recorded at all three visits.	The relative risk of GD was 0.75 (95% CI [0.57, 0.98], *p* = 0.039). Rates of insulin-treated GD were also significantly reduced: (RR = 0.43, 95% CI [0.24, 0.78], *p* = 0.006); prematurity (OR = 0.29, 95% CI [0.11, 0.77], *p* = 0.013); gestational weight gain at 24–28 and 36–38 weeks of gestation, *p* = 0.022 and *p* = 0.037, respectively); emergency Cesarean section (OR = 0.30, 95% CI [0.14, 0.63], *p* = 0.001); perineal trauma (OR = 0.21, 95% CI [0.12, 0.36], *p* = 0.001); ITUs (OR = 0.41, 95% CI [0.26, 0.64], *p* = 0.001) and low-weight newborns and macrsomia (RR = 0.21, 95% CI [0.08, 0.54], *p* = 0.001) and (RR = 0.19, 95% CI [0.07, 0.57], *p* = 0.003), respectively.	Early nutritional intervention with supplemented MD reduces the incidence of GD and improves several maternal and neonatal outcomes.
Al Wattar et al. (35)	The control group received dietary advice according to UK national recommendations for antenatal care, whereas the intervention group attended three individual and two group sessions to promote a Mediterranean-style diet and were provided with nuts (30 g/day of walnuts, hazelnuts, and almonds) and EVOO (0.5 l/week). To assess adherence to the diet, a validated FFQ for md and a modified short questionnaire (ESTEEM Q) were used. To evaluate GD, an oral glucose tolerance test was performed on all participants.	A simple, individualized, Mediterranean-style diet during pregnancy has the potential to reduce the chances of GD by 35% (OR = 0.65, 95% CI [0.47, 0.91], *p* = 0.01) and reduce gestational weight gain (mean 6.8 vs. 8.3 kg; OR = –1.2 kg, 95% CI [–2.2, –0.2], *p* = 0.03), compared to routine prenatal care.	A simple, individualized, Mediterranean-style diet has the potential to reduce weight gain and GD risk.
Saunders et al. (36)	The degree of adherence to md during pregnancy was assessed with an FFQ based on nine dietary criteria and with a scale constructed by Trichopoulou, both administered in the days after delivery. Preterm birth was defined as any birth that occurred before the 37th week of gestation, while birth weight was extracted from pediatricians’ records at birth.	Among women who were overweight or obese before pregnancy, the risk of preterm labor was significantly lower for those who followed MD during pregnancy (OR = 0.7, 95% CI [0.6, 0.9], *p* < 0.01).	Adherence to MD in the Caribbean population may decrease the risk of preterm birth in overweight and obese pregnant women.
Tobias et al. (37)	Questionnaires were distributed every 2 years (from 1991 to 2001) to update lifestyle characteristics and health-related outcomes. In that space of time, in addition to the main questionnaire, an FFQ was added every 4 years. A medical diagnosis of GD was verified by self-report in each biennial questionnaire up to 2001.	MD was associated with a 24% lower risk of developing GD during pregnancy (RR = 0.76, 95% CI [0.60–0.95], *p* = 0.004). The antihypertensive diet, associated with a 34% lower risk (RR = 0.66, 95% CI [0.53, 0.82], *p* = 0.0005) and the alternative healthy dietary pattern, with a 46% lower risk (RR = 0.54, 95% CI [0.43, 0.68], *p* < 0.0001).	Adherence to healthy dietary patterns during pregnancy is significantly associated with a lower risk of GD.

Nine studies (18–20, 28, 29, 34–37), found positive maternal effects related to adherence to MD during pregnancy.

#### Gestational Diabetes

A total of five studies (18, 28, 29, 35, 37) obtained found that MD acts as a protective factor against the development of GD.

In three studies (18, 29, 37), MD was supplemented with EVOO and nuts. Two articles found that high adherence to MD was associated with a 65% lower risk of GD (*p* = 0.002) (18) and (*p* = 0.039) (18, 29). MD supplemented with EVOO and nuts in pregnant women with metabolic risk (obesity, arterial hypertension, or hypertriglyceridemia) was also found to lead to a 35% lower likelihood of developing GD if their adherence to MD was high (*p* = 0.01) (35).

Two studies analyzed the effect of MD consumption before pregnancy. One of the studies showed a decrease of 24% in the risk of developing GD (*p* = 0.004) when women followed an MD nutritional pattern before pregnancy (33). In addition, one study (28) found a protective effect of MD and low consumption of meat and derivatives on the development of GD (*p* = 0.028 for high adherence to MD and *p* = *0.005* for very high adherence) (28).

#### Healthy Lifestyle

Four studies (19, 20, 29, 35) analyzed the relationship between MD consumption during pregnancy and the healthy lifestyles during pregnancy. Results suggested that mothers with adherence to MD gained less gestational weight (29, 35). Also, women with a strong preconception adherence to MD gained less weight during pregnancy (20). A cross-sectional study conducted by Flor-Alemany et al. (19) showed that greater adherence to MD was associated with better sleep quality during weeks 16–34 of gestation, especially among sedentary pregnant women (*p* < 0.05).

#### Complications of Childbirth

Two studies (29, 36) associated MD consumption with reduced complications during childbirth.

First, in the cohort study conducted by Saunders et al. (36), it was hypothesized that adherence to MD decreases the risk of preterm birth but they only found relevant results in overweight or obese women (*p* < 0.01).

In addition, in the second study (29), it was found that women who had followed an MD pattern were less likely to give birth by emergency Cesarean section as well as a lower probability of perineal trauma during childbirth (both, *p*s = 0.001). This reduction could be due to improvements in the evolution of childbirth or also to the reduction in the number of newborns with macrosomia (29).

#### Urinary Tract Infections

In two studies, high adherence to MD was found to reduce the risk of UTIs during pregnancy. This relationship (*p* = 0.001) was found in the research of Assaf-Balut et al. (18, 29), and this result may be due to the relationship between MD, inflammation, and immunomodulation. This effect is possibly due to the presence of some food components, such as phenolic compounds and oleic acid (29).

#### Fetal Growth

Finally, one study (37) found a significant association between maternal adherence at the beginning of pregnancy to a high content of fish and EVOO and a dietary pattern low in meat (characteristic food of the Mediterranean diet), with increased fetal growth. An increase in embryonic volume was also found, with percentages of 20.4% at 7 weeks and 14.4% at 11 weeks (34).

### Perinatal Outcomes

Table 7 shows the results of the studies analyzed regarding perinatal benefits. A total of seven studies (18, 21, 29–33), showed a statistically significant relationship between adequate maternal adherence to MD and improvements in perinatal health.

**TABLE 7:** Perinatal outcomes.

References	Objective	Outcomes	Conclusion
Assaf-Balut et al. (18)	The women of the intervention group attended two group sessions where they were instructed to improve the consumption of EVOO and nuts and they received 10 L of EVOO and 2 kg of pistachios in each session. The control group received basic dietary guidelines and was told to limit all types of fat intake (including < 3 servings/week of walnuts and < 40 ml/day of EVOO). To assess lifestyle and diet, the semi-quantitative Diabetes Nutrition and Complications Trial (DNCT) questionnaires and the MEDAS questionnaires were used, which were administered on 3 different visits. Blood pressure, height, weight, gestational weight gain, and BMI were assessed and recorded at all three visits.	There was a linear association between high, moderate, and low adherence and a lower risk of GD (OR = 0.35, 95% CI [0.18, 0.67], *p* = 0.002), UTIs (OR = 0.19, 95% CI [0.07, 0.52], *p* = 0.001), prematurity (OR = 0.30, 95% CI [0.13, 0.72], *p* = 0.007) and low-weight newborns (OR = 0.36, 95% CI [0.17, 0.77], *p* = 0.009).	High adherence to the six predefined dietary targets at the end of the first trimester is associated with a reduction in the risk of GD, UTIS, prematurity, and low birth-weight infants.
Chatzi et al. (21)	Mothers reported their diet from the time of their last menstrual period using a validated semiquantitative FFQ. The overall dietary pattern was examined using the Trichopoulou score. In addition, weight, height, abdominal perimeter, thickness of subscapular skin folds and triceps, systolic and diastolic blood pressure, and lipid, leptin, and adiponectin levels of children were measured	High maternal adherence to MD was associated with a lower BMI score in the offspring of 0.14 units (95% CI [–0.15, –0.13]), abdominal perimeter at 0.39 cm (95% CI [–0.64, –0.14]), and the sum of the skinfold thickness by 0.63 mm (95% CI [–0.98, –0.28]). The authors also observed lower systolic (–1.03 mmHg, 95% CI [–1.65, –0.42]) and diastolic blood pressure (–0.57 mmHg, 95% CI [–0.98, –0.16]) in childhood.	Increased adherence to MD during pregnancy may protect against excess cardiometabolic risk in childhood.
Assaf-Balut et al. (29)	The women of the intervention group attended two group sessions where they were instructed to improve the consumption of EVOO and nuts and they received 10 L of EVOO and 2 kg of pistachios in each session. The control group received basic dietary guidelines and was told to limit all types of fat intake (including < 3 servings/week of walnuts and < 40 ml/day of EVOO). To assess lifestyle and diet, the semi-quantitative Diabetes Nutrition and Complications Trial (DNCT) questionnaires and the MEDAS questionnaires were used, which were administered on 3 different visits. Blood pressure, height, weight, gestational weight gain, and BMI were assessed and recorded at all three visits.	The relative risk of GD was 0.75 (95% CI [0.57, 0.98], *p* = 0.039). Rates of insulin-treated GD were also significantly reduced: (OR = 0.43, 95% CI [0.24, 0.78], *p* = 0.006); prematurity (OR = 0.29, 95% CI [0.11, 0.77], *p* = 0.013); gestational weight gain at 24–28 and 36–38 weeks of gestation (*p* = 0.022 and *p* = 0.037, respectively); emergency Cesarean section (OR = 0.30, 95% CI [0.14, 0.63], *p* = 0.001); perineal trauma (OR = 0.21, 95% CI [0.12, 0.36], *p* = 0.001); UTIs (OR = 0.41, 95% CI [0.26, 0.64], *p* = 0.001); and low-weight newborns and macrsomia (OR = 0.21, 95% CI [0.08, 0.54], *p* = 0.001) and (OR = 0.19, 95% CI [0.07, 0.57], *p* = 0.003), respectively).	Early nutritional intervention with supplemented MD reduces the incidence of GD and improves several maternal and neonatal outcomes.
Martínez-Galiano et al. (30)	A paired case study (children with low birth weight) and controls (normal weight children) was conducted. For the dietary evaluation, an FFQ was used for the previous year’s intake, while three indices were used to evaluate adherence to MD: Predimed, Trichopoulou, and Pangiotakos.	Adherence to MD and daily intake of 5 g of EVOO was associated with a lower risk of low birth weight (OR = 0.59, 95% CI [0.38, 0.98]).	Adherence to MD and EVOO intake is associated with a reduced risk of underweight newborns.
Melero et al. (31)	Prospective analysis of the prevention study of GD of St. Carlos [18, 28]. After delivery, a follow-up was carried out for 2 years, in which both the control group and the intervention group received the same recommendations, and the same questionnaires were used for dietary evaluation.	Adherence to MD enriched with EVOO and pistachios during pregnancy was associated with children of mothers with pregestational BMI < 25 kg/m^2^ and normal glucose tolerance (NGT) having a lower risk (OR (95% CI) of severe events requiring hospitalization due to bronchiolitis or asthma (OR = 0.75, CI [0.58, 0.98] and (OR = 0.77, CI [0.59, 0.99], respectively) or other diseases requiring antibiotic or corticosteroid treatment (OR = 0.80, CI [0.65, 0.98] and (OR = 0.73, CI [0.59, 0.90], respectively) (all *ps* < 0.05).	A nutritional intervention based on MD during pregnancy is associated with a reduction in childhood hospital admissions, especially in women with pregestational BMI < 25 kg/m^2^ and normal glucose tolerance.
Cánovas-Conesa et al. (32)	A case study (children with gastroschisis) and controls (healthy children) was conducted. At the time of diagnosis, each case completed a validated FFQ of the diet consumed during pregnancy.	A maternal diet rich in oleic acid (OR = 0.79, 95% CI [0.65, 0.97]) and plant products (OR = 0.70, 95% CI [0.48, 1.00]) was associated with preventing the risk of vascular occlusion of the omphalomesenteric arteries, decreasing the risk of gastroschisis.	A maternal diet rich in oleic acid and plant products can prevent vascular risk of the omphalomesenteric arteries, reducing the risk of gastroschisis.
Steenweg-de Graaff et al. (33)	Nutritional intake for the last 3 months at the beginning of pregnancy was assessed using a modified version of an FFQ. In addition, mothers were asked to complete the Child Behavior Checklist for Young Children (CBCL), which serves to measure the degree of problematic behavior of children.	MD was negatively associated (OR = 0.90, 95% CI [0.83, 0.97], *p* = 0.006) and the traditional Dutch diet was positively associated with child externalizing problems (OR = 1.11, 95% CI [1.03, 1.21], *p* = 0.011). No diet was associated with internalizing problems.	Both low adherence to MD and high adherence to the traditional Dutch diet during pregnancy are associated with an increased risk of externalizing problems in the child.

#### Birth Weight

Three studies (18, 29, 30) found a relationship between maternal adherence to MD and birth weight. In particular, it was found that MD reduced the probability of low birth weight (18, 29) and that the EVOO component and the presence of macrosomia was essential to control the low birth weight (35) because the risk of macrosomia is inversely associated with diets of low glycemic index, a characteristic of MD. The risk of newborns with low weight is a consequence of placental insufficiency. In this sense, MD supplemented with EVOO and nuts could play a fundamental role in improving placental flow and, therefore, decreased intrauterine growth retardation (29).

#### Prematurity

Maternal adherence to MD was associated with a significant reduction in the rates of preterm infants (18, 29).

#### Gastroschisis

In a study by Cánovas-Conesa et al. (32), it was observed that adherence to MD, rich in EVOO and vegetables, helped to prevent the risk of vascular occlusion and risk of gastroschisis.

#### Problems in Childhood

The relationship between maternal adherence to MD and health in children’s development was analyzed in three studies (21, 31, 33). First, the cohort study of Chatzi et al. (21) revealed that increased adherence to MD at early gestation was associated with lower adiposity levels (BMI 0.14 units lower, arm crease thickness 0.63 mm lower, and abdominal circumference 0.39 mm lower), leptin (reflecting lower fat and lower triglyceride levels in childhood) and lower systolic and diastolic blood pressure in children. According to the authors, this may be due to the effect of MD’s low glycemic index and its high antioxidant content, which can lead to better fetal glucose metabolism and better metabolic function and, finally, influence the individual’s susceptibility to weight gain in childhood (21).

In addition, in the research conducted by Steenweg-de Graaff et al. (33), an association was found between the consumption of MD during pregnancy and a lower risk of developing behavioral problems in childhood, specifically, externalizing problems (attention problems and aggressive behavior) (*p* = 0.006), whereas the traditional Dutch diet favored the development of these problems (*p* = 0.011) by Steenweg-de Graaff et al. (33).

Finally, the study of Melero et al. (31) found a relationship between maternal intake of MD and reduced risk of hospitalization in children up to 2 years of age. However, this relationship was stronger in women with a pregestational BMI < 25 kg/m^2^ and normal glucose tolerance (*p* < 0.05) (31).

## Discussion

The objective of this study was to conduct a systematic review of the literature to study the effects of the MD during the gestational period both on the mother and the fetus. A total of 14 studies were analyzed. The results showed that the MD was associated with the reduction of some pathologies of pregnancy, such as GD, overweight or obesity, complications of childbirth, UTIs, alterations of fetal growth, and sleep quality; as well as perinatal problems, including birth weight, prematurity, gastroschisis, and other childhood problems. These results may be due to the relationship between MD, inflammation, and immunomodulation. This effect is possibly a consequence of the presence of some food components, such as phenolic compounds and oleic acid (29). Some previous studies have suggested that some foods such as nuts or olive oil may facilitate weight loss and may have potential effects on insulin sensitivity and inflammation (38). Also, the MD has potential antioxidant effect given their high levels of lutein, β-carotene, and γ-tocopherol (39) improving the inflammatory cytokine profiles.

Likewise, the results of this study show that MD favors a decrease in complications during pregnancy, as it is related to a lower risk of preterm delivery (32), emergency Cesarean sections, and perineal trauma (29). Also, adherence to MD promotes adequate fetal growth (34) because this type of diet provides the nutrients necessary for the correct development of the fetus (34). As for the newborn, MD intake during pregnancy has been shown to be a protective factor linked to a decrease in the number of hospitalizations (31), a lower risk of gastroschisis (32), a decrease in attention problems and aggressiveness (33), as well as a lower risk of developing cardiometabolic complications, such as hypertension or obesity (21), a lower risk of prematurity (18, 29), and adequate birth weight (18, 29, 30).

These results are similar to those obtained in other systematic reviews. For example, Biagi et al. (40) and Amati et al. (41) found a positive effect between adherence to MD during pregnancy and children’s health. Hassani Zadeh et al. showed that following an MD pattern during pregnancy is beneficial both for the mother and the offspring, and also showed that the MD can potentially decrease the risk of suffering from atopia in the offspring and GD in the mother (42), results that are highly consistent with our study. In particular, MD is associated with a lower incidence of asthma/allergic diseases in childhood, although further studies would be needed to clarify this relationship (38). The beneficial effects of MD had also been associated with health parameters, including improved glycemic control, a reduction in insulin use during pregnancy, a decreased risk of newborns with macrosomia (43), and better control of blood pressure. Eating patterns before and during pregnancy characterized by a high intake of fruits, vegetables, legumes, whole grains, nuts, olive oil, and a low intake of red meat and processed meats have been associated with an increased risk of hypertension during pregnancy. Although the biological mechanisms by which MD plays a protective role are not precisely known, these findings could indicate that the consumption of unsaturated fatty acids and micronutrients as well as some bioactive compounds make this diet help prevent complications during pregnancy (44).

Due to the beneficial effects of the MD, some authors highlight the need to recover the MD pattern in the general population and, in particular, in pregnant women, to avoid overweight and obesity, problems during pregnancy, as well as to achieve an adequate weight gain, as all this is related to overweight and the increase in adiposity in the offspring (40, 41, 44, 45).

The present work presents several limitations that require future areas of research. First, the wide variety in the design of the included studies, the variability of the strength of association in different health outcomes, and the presence of studies that used a small sample size limit the conclusions. In addition, the search only included publications in English and Spanish; therefore, not all current evidence to date may be represented. Thirdly, the data sources used for this review were PubMed, Medline, and the Cochrane Library. Fourthly, the intervention was performed at different times of gestation, which may affect the robustness of the results and conclusions. Also, there is a bias of quality and selection of articles that, despite having been reduced to the maximum, is still inherent in this type of study. And finally, there is high variability in the type of intervention performed, in the method of collecting data, and in the foods that constitute MD in each region, which can make it difficult to compare their effectiveness. However, this study has strengths; for example, it is one of the few reviews existing in the literature, where the relationship of the MD is analyzed in an applied way not only in pregnancy, but also in the offspring. This study provides also evidence about the beneficial effect of the MD in the reduction of some pathologies of pregnancy including: sleep problem, gastroschiis, alteration in fetal growth and prematurity. In addition, we examined the quality of studies using three independent tool. We found that most studies (80%) described main areas of bias in controlled clinical studies including: eligibility criteria, allocation, randomization, measurement of drop outs, measures of at least one key outcome, results between group. However, few studies were described as double-blind or the blinding method was inappropiated. Future studies should be carried out controlling the ROB of studies. Finally, future longitudinal studies should be carried out with a large sample to obtain strongly conclusive data evidencing the need to promote adherence to the MD in this population, working not only with pregnant women but with the family as a whole. In this regard, more studies on health promotion and prevention are needed to improve the health of pregnant women and their families.

## Conclusion

The MD is optimal to ensure an adequate supply of nutrients during pregnancy. It provides all the energy and nutrients required to allow fetal growth and development, protecting from the development of obstetric pathologies including complications of childbirth, infections and alteration in fetal growth. The MD diet also can prevent some maternal complications such as diabetes, sleep quality and overweight or obesity. Given the available evidence of changes affecting women’s health during pregnancy, it is necessary to follow an adequate diet during gestation. Therefore, strategies to promote adherence to MD can be of considerable importance for public health given that MD may play a protective role in maternal and fetal health during the gestational period.

## Data Availability Statement

The original contributions presented in the study are included in the article/supplementary material, further inquiries can be directed to the corresponding authors.

## Author Contributions

NR-R and AZ-M: conceptualization. NR-R, AZ-M, and MS-S: methodology. VS, IH-C, MS-S, and JH-S: validation. NR-R: formal analysis and writing—original draft preparation. NR-R, AZ-M, MS-S, JH-S, VS, and IH-C: investigation. AZ-M: data curation and supervision. NR-R, AZ-M, MS-S, and VS: writing—review and editing. NR-R, AZ-M, MS-S, JH-S, and VS: visualization. All authors have read and agreed to the published version of the manuscript.

## Conflict of Interest

The authors declare that the research was conducted in the absence of any commercial or financial relationships that could be construed as a potential conflict of interest.

## Publisher’s Note

All claims expressed in this article are solely those of the authors and do not necessarily represent those of their affiliated organizations, or those of the publisher, the editors and the reviewers. Any product that may be evaluated in this article, or claim that may be made by its manufacturer, is not guaranteed or endorsed by the publisher.
